# Assessment of cecal microbiota modulation from piglet dietary supplementation with copper

**DOI:** 10.1186/s12866-023-02826-9

**Published:** 2023-03-31

**Authors:** Ana Carolina Laureano Paganin, Paulo Sérgio Monzani, Marcelo Falsarella Carazzolle, Raquel Bighetti Araujo, Ricardo Gonzalez-Esquerra, Douglas Haese, João L Kill, Graziela Silva Rezende, César Gonçalves de Lima, Iran Malavazi, Caio César de Melo Freire, Anderson Ferreira da Cunha

**Affiliations:** 1 Departamento de Genética e Evolução, Universidade Federal de São Carlos, São Carlos, SP Brasil; 2 Faculdade de Zootecnia e Engenharia de Alimentos, Departamento de Medicina Veterinária, Universidade de São Paulo, Pirassununga, SP Brasil; 3 Departamento de Genética, Evolução, Microbiologia e Imunologia, Universidade de Campinas, Campinas, SP Brasil; 4 Novus International, Indaiatuba, SP Brasil; 5 Centro de Tecnologia Animal Ltda, Espirito Santo – ES, Domingos Martins, Brasil

**Keywords:** 16S rRNA gene, Microbiota, Swine, Copper, Feed supplement, Animal nutrition

## Abstract

**Background:**

Swine production expanded in the last decades. Efforts have been made to improve meat production and to understand its relationship to pig gut microbiota. Copper (Cu) is a usual supplement to growth performance in animal production. Here, two performance studies were conducted to investigate the effects of three different sources of Cu on the microbiota of piglets. A total of 256 weaned piglets were randomly allocated into 4 treatments (10 replicates per treatment of 4 piglets per pen in Trial 1 and 8 replicates of 3 piglets per pen in Trial 2). Treatments included a control group (fed 10 mg/kg of Cu from CuSO_4_), a group fed at 160 mg/kg of Copper (II) sulfate (CuSO_4_) or tri-basic copper chloride (TBCC), and a group fed with Cu methionine hydroxy analogue chelated (Cu-MHAC) at 150, 80, and 50 mg/kg in Phases 1 (24–35 d), 2 (36–49 d), and 3 (50–70 d), respectively. At 70 d, the cecum luminal contents from one pig per pen were collected and polled for 16 S rRNA sequencing (V3/V4 regions). Parameters were analyzed in a completely randomized block design, in which each experiment was considered as a block.

**Results:**

A total of 1337 Operational Taxonomic Units (OTUs) were identified. Dominance and Simpson ecological metrics were statistically different between control and treated groups (P < 0.10) showing that different Cu sources altered the gut microbiota composition with the proliferation of some bacteria that improve gut health. A high abundance of *Prevotella* was observed in all treatments while other genera were enriched and differentially modulated, according to the Cu source and dosage. The supplementation with Cu-MHAC can modify a group of bacteria involved in feed efficiency (FE) and short chain fatty acids (SCFA) production (*Clostridium XIVa, Desulfovibrio*, and *Megasphera*). These bacteria are also important players in the activation of ghrelin and growth hormones that were previously reported to correlate with Cu-MHAC supplementation.

**Conclusions:**

These results indicated that some genera seem to be directly affected by the Cu source offered to the animals. TBCC and Cu-MHAC (even in low doses) can promote healthy modifications in the gut bacterial composition, being a promising source of supplementation for piglets.

**Supplementary Information:**

The online version contains supplementary material available at 10.1186/s12866-023-02826-9.

## Background

The microbiota in the mammalian gastrointestinal tract (GIT) has about 10^14^ bacteria [1]. Species interact and contribute to the metabolism in processes such as energy acquisition from food [2]. The cecum is one of the most microorganisms diverse to the gastrointestinal segments. The high prevalence of fermentative microorganisms is supported by the meaningful function of the large intestine (cecum and colon), in absorbing short-chain fatty acids (SCFA), in addition to vitamin K, B7, potassium, and sodium [3]. The large intestine contains gut-associated lymphoid tissues (GALT) that are important sites for intestinal effector lymphocyte generation, thus contributing to adaptive immune responses [4].

The complex gut microbiota influences intestinal homeostasis and immunologic process, for example, the anti-inflammatory role [5]. Some factors such as age, diet, and drugs are known to be microbiota composition modifiers [6]. Several GIT microbiomes have been studied to elucidate their role in the modulation of gut health, such as human [7, 8], bovine [9], chicken [10, 11], and swine [12, 13]. Swine is the second most widely eaten meat worldwide, after poultry [14], so efforts to improve production are essential to develop the industries and increase the availability of this food to the population. In the piglet post-weaning transition, GIT microbiota changes [15] can increase the incidence of diarrheal infection [16, 17]. To prevent diseases and improve feed efficiency, antibiotics are still widely used. However, antibiotics are banned in several countries. Supplements could be used as an alternative, but studies comparing the benefits and synergistic and adverse effects of alternative supplements must be thoroughly studied [16, 18].

The feed supplements usually administered are prebiotics, plant extracts, and minerals, among others [19, 20]. Trace minerals are required for animal development while presenting antimicrobial properties when used in doses higher than the nutritional requirements [21]. For instance, zinc (Zn) is fundamental to biological processes in mammals [21], and its supplementation as zinc oxide (ZnO) has decreased diarrhea cases, improving piglet growth [19, 20]. Copper (Cu) is crucial to many metalloenzymes, cellular protection against oxidative stress and metabolic reactions [19], and it has been used for its potential antibiotic properties since ancient times [22]. The uptake of Cu seemed to be regulated by different transporters and transport mechanisms and depends on the source with which it is associated [23]. A study on supplementation with tribasic copper chloride (TBCC) and copper sulfate (CuSO_4_) showed that both improved performance in weaning pigs [24]. However, evidence indicates that TBCC has fewer adverse effects on animals than CuSO_4_ [19].

Despite the benefits, some undesirable effects are associated with supra-nutritional levels of Cu (125 to 250 mg/kg) depending on the source (i.e., antagonisms, vitamin oxidation, high excretion, presence of contaminants, etc.). The organic source Copper methionine hydroxy analogue chelated (Cu-MHAC) has been demonstrated to be more bioavailable, so lower concentrations are needed for similar growth promoter effects [25]. In addition, piglets fed lower levels of Cu through Cu-MHAC had an improvement in general performance than when fed CuSO_4_ and TBCC. The results also suggested an increase in mRNA expression of ghrelin and serum growth hormone (GH) levels in the animals [26]. To add to the knowledge on the mode of action of Cu sources, this study investigated the cecal microbiota composition of piglets after dietary supplementation with Cu-MHAC, CuSO_4,_ or TBCC.

## Methods

### Animal and sample collection

The experimental procedures followed Gonzalez-Esquerra et al. [26]. Two trials of equal design were conducted sequentially in the same open-side barn piglet facility with slatted-floor pens. The barn was not cleaned before the trials to simulate common adverse conditions. A total of 256 commercially acquired Agroceres PIC piglets weaned at 24 ± 2 d were used. Trial 1 (summer) included 160 piglets (80 barrows and 80 guilts) weighted 5,43 ± 0,90 kg and Trial 2 (spring) included 96 piglets (48 barrows and 48 f) weighted 4,73 ± 0,95 kg. Pigs were allocated to four treatments in a completely randomized block design with ten replicates per treatment. We used four pigs per replicate in Trial 1 and three pigs per replicate in Trial 2 in four treatments. All groups were fed corn, soybean meal, and dairy by products based diets and submitted to dietary treatments from 24 to 70 days as follows: (i) the Control received 10 mg/kg of Cu from copper sulfate, (ii) the second group was supplemented with 160 mg/kg Cu from CuSO_4_, (iii) the third group supplemented with 150 (from 24 to 35 d), 80 (36 to 49 d) and 50 mg/kg Cu (50 to 70 d) from Cu-MHAC, and (iv) the fourth group supplemented with 160 mg/kg Cu from TBCC. Zinc oxide was included during the first 2 phases post-weaning at 2,200 and 1,500 mg/kg as commonly used in piglets in Brazil. Feed and water were provided ad libitum throughout the entire experimental period and the diets contained antibiotics (halquinol at 200 g/ton in all phases and amoxicillin at 255 g/ton in Phase 2 only). At 70 d of age, animals were sacrificed and the cecum luminal contents from one pig per pen (72 animals) were collected, snap-frozen in liquid nitrogen, and stored at -80 ºC until analyses.

### DNA extraction and 16 S rRNA sequencing

The genomic DNA purification, quantification, sequencing, read processing, and phylogenetic analyses were performed as previously described (9). Briefly, genomic DNA from each sample was purified using QIAamp Fast DNA Stool Mini Kit (QIAGEN, Hilden, Germany) following the manufacturer. Then, DNA quality was evaluated by agarose gel electrophoresis and quantified using the NanoVue Plus spectrophotometer (GE Healthcare, Marlborough, USA). After quantification, all samples were diluted at 50 ng/µL. Four pools per treatment (two pools per trial) were produced using the same volume (5 µL) of 4 samples for Trial 1 and 3 samples for Trial 2. The pooled samples from the cecum were used to amplify approximately 460 bp of the 16 S ribosomal RNA by PCR using specific primers V3 and V4 (Klindworth et al., 2013). PCR products were used to build the metagenomics library for sequencing using MiSeq Reagent kit v3 (600 cycles) (Illumina Inc., San Diego, California, USA). The sequencing of partial 16 S ribosomal RNA was performed by next-generation sequencing using the Illumina MiSeq platform that produced thousands of 300 bp paired-end reads (2 × 300 bp) for each library.

### Bioinformatics analysis

The reads from each pool were analyzed on USEARCH (version 10.0.240) [27]. The pair of reads were merged with a minimum of 200 bp and filtered by the quality and unique abundance. This data was used for the rarefaction curve, alpha diversity calculation, and Venn diagram (http://bioinformatics.psb.ugent.be/webtools/Venn/). We tested a total of eleven alpha metrics regarding diversity (Richness, Chao 1, Shannon, Jost, and Jost 1) and evenness (Simpson, Dominance, Equitability, Robbins, Berger, and Parker). The phylogenetic categories found were compared to the Ribosomal Data Project (RDP) classifier [28] with 0.80 to a cutoff on the USEARCH.

### Statistical analyses

Data were analyzed in a completely randomized block design using PROC GLM of SAS 9.3 (SAS Inc., NC, 2011) with each experiment used as a block. Differences among means were compared by Tukey’s least significant difference. Orthogonal contrasts were used to test relevant comparisons which included the effect of Cu-MHAC vs. feeding inorganic Cu (CuSO_4_ and TBCC), CuSO_4_ vs. TBCC, and nutritional vs. supra-nutritional Cu levels (Control vs. Others). Differences among the groups were considered significant when p-value < 0.05 while p-values ranging from 0.05 to 0.10 was denoted as a statistical trend since they were also biologically relevant, as previously demonstrated [29]. Analysis of variance (ANOVA) was used to determine if the means of the treatments were different. Non-parametric statistics were applied to the data that did not meet the assumptions of the statistical model using the Freedman test. The hierarchical cluster of the heatmap made with pvclust (version 2.0.0) [30] aimed at grouping treatments and OTUs, testing with 10,000 interactions.

### Data availability

16 S sequencing data for the control and all treatments have been submitted to the NCBI under BioProject ID: PRJNA798269 and in the following link: https://www.ncbi.nlm.nih.gov/bioproject/PRJNA798269.

## Results and discussion

### Treatments with different copper sources and levels were related to the modification of the microbial composition in piglets

We sequenced 16 pooled samples from the cecum of 256 animals (four replicates of each treatment: Control, Cu-MHAC, CuSO_4_, and TBCC). Pooled samples were used to reduce individual variabilities focusing on the modifications produced by the treatments. Our analyses revealed that 1,858,722 high-quality sequences were obtained and distributed in 1,337 Operational Taxonomic Units (OTUs) (Table S1). Although the number of reads and OTUs was different among samples, the rarefaction plots (Fig. S1a) of the variables (OTUs and reads), and (Fig. S1b) of the Chao1 (alpha diversity metric), with the data subsampled (± 10,000 per sample), showed that all curves were rarefied, so the collected data were representative of the sampled diversity. Beta diversity (Bray-Curtis dissimilarity) analysis indicated that the diversity of the samples collected in the same trial was more similar, considering the bacterial community for each one, as observed in Principal component analysis (PCA) in Fig. S1c. The impact of the treatment can be considered lower, considering this beta diversity metric.

Eleven alpha diversity metrics analyzed were divided into richness and equitability categories. Table 1 shows the mean of each metric by treatment, the coefficient of variation of the mean (CVmean), and the statistics of the mean. The uniformity and equitability index measured by Buzas-Gibson, Robbins, and equitability metrics showed a significant statistical difference between the two trials (used as blocks). The gut environment has great importance on the microbiota composition and microbial metabolism. Sometimes, a small change in environmental factors such as temperature or pH could lead to drastic alterations [31]. Alterations could be related to the season in which that trial was conducted. Considering the effect of treatment, the mean of treatments of Dominance and Simpson diversity metrics tended to indicate differences (P < 0.10) in the analysis of variance (ANOVA), pointing to an effect of high levels of Cu on the evenness of the microorganisms in the gut (as seen by contrasting Control vs. Others). The decrease in Simpson and increase in Dominance diversity indexes indicate that Cu-MHAC tended to increase the microorganism diversity compared to TBCC (P < 0.10 by contrast) (Table 1).

**Table 1:** Alpha diversity metrics means by treatment, ANOVA, and orthogonal contrasts analysis

Treatment	Alpha diversity metrics										
	Berger Parker	Buzas Gibson	Chao 1	Dominance	Equitability	Jost	Jost 1	Richness	Robbins	Shannon	Simpson
Control	0.1190	0.0011	723.2250	0.9692	0.6827	49.1000	88.9250	722.0000	0.1607	1.9475	0.0306
Cu-MHAC	0.0998	0.0008	199.1000	0.9750	0.6957	59.6000	104.7500	798.0000	0.1400	2.0150	0.0250
CuSO_4_	0.0968	0.0011	745.4000	0.9787	0.6940	59.4500	101.0750	744.2500	0.1477	1.9900	0.0212
TBCC	0.1212	0.0008	796.0500	0.9700	0.6832	51.8500	96.7000	795.0000	0.1367	1.9825	0.0301
CV_mean_ (%)	30.7300	38.1200	8.7000	0.5000	3.2600	21.6200	17.0200	8.7400	17.6000	3.6300	17.9800
***P*** **-value for ANOVA**											
Trial	0.5294	0.0167**	0.1433	0.9306	0.0689*	0.4072	0.2818	0.1436	0.0434**	0.2635	0.8741
Treatment	0.6451	0.4934	0.3300	0.0908*	0.7711	0.5155	0.5890	0.3313	0.5784	0.6298	0.0892*
***P*** **-value for Orthogonal contrasts**											
Control vs. Others	0.5141	0.3868	0.1666	0.0920*	0.5374	0.2762	0.2410	0.1671	0.2219	0.2703	0.0989*
Cu-MHAC vs. (CuSO_4_ + TBCC)	0.5177	0.2288	0.2272	0.0916*	0.7495	0.6191	0.9732	0.2281	0.5642	0.8466	0.0867*
CuSO_4_ vs. TBCC	0.3860	0.7304	0.9495	0.1772	0.4476	0.3765	0.5084	0.9505	0.8616	0.5370	0.1676

Significance level was considered p-value < 0.05**, and p-value from 0.05 to 0.10 indicated a statistical trend*. Coefficient of variation of the mean (CV_mean_)

### Treatments with different copper sources regulate piglets’ gut microbiota

**Fig. 1:**
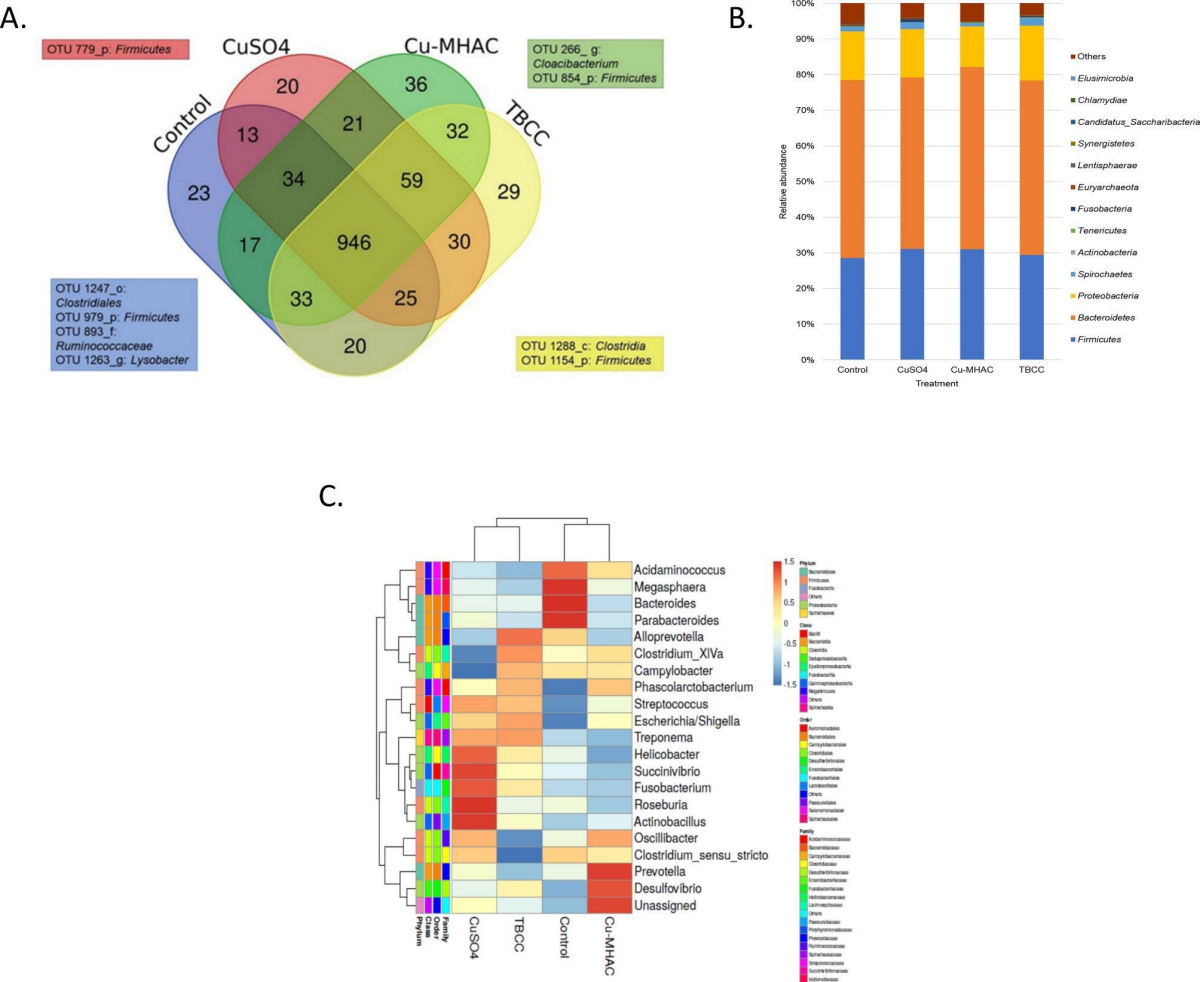
Taxonomic distribution of treatments with different copper supplementations. Venn diagram of intersections and exclusive numbers of OTUs from the Control, CuSO_4_, Cu-MHAC, and TBCC treatments. The identified OTUs are in at least two samples (**A**). Phylum composition relative abundance by treatment (**B**). Heatmap showing the twenty most prevalent genera and the “Unassigned” bacteria by treatment. The color gradient indicates the lowest (blue) and highest (red) abundances. The taxonomic levels (domain, phylum, class, order, and family) were indicated in Table S2. The clustering was made using the Euclidean distance and complete linkage method in the web tool ClustVis [78], and analyzed by pvclust Fig. S2 [30] (**C**)

A total of 946 OTUs were shared among treatments, while some OTUs were exclusive to one treatment (Fig. 1A). The Control treatment presented 23 exclusive OTUs, CuSO_4_ presented 20, Cu-MHAC presented 36, and TBCC presented 29. Although some of these OTUs were from the *Firmicutes* phylum, they were represented by different OTUs related to a specific treatment, indicating that they may represent different species of this phylum. *Firmicutes* is abundant in swine gut microbiota [32, 33], comprising the class *Clostridia* that embraces strict anaerobe genera as *Clostridium, Ruminococcus, Dorea*, and *Eubacterium*. These members can be associated with gut homeostasis, immune system, or recognized pathogens on a global scale, like *Clostridium difficile* [34–37].

The genus *Lysobacter* (OTU 1,263), the order *Clostridiales* (OTU 1,247), and the family *Ruminococcaceae* (OTU 1,247) were only found in the Control group. Several species of *Lysobacter* are associated with antibiotic sources and with cooper resistance [38], while *Ruminococcaceae* has been shown as adjuvants to immune checkpoint inhibitors and negatively associated with the presence of endotoxin [39]. Species from the *Clostridiales* order were reported to attenuate inflammation and allergic diseases. In addition to the OTU 1,247 found exclusively in the control group, we found a specific genus known as *Clostridium cluster XIVa*. This genus plays an important role in intestinal homeostasis, and it was increased with TBCC treatment, as later discussed (Table 2).

*Cloacibacterium* (OTU 266) was found exclusively in animals treated with Cu-MHAC. *Cloacibacterium* has four recognized species [40] and it was statistically more abundant in unreactive (“health”) ileocecal lymph nodes, in comparison to pathologically changed nodes of slaughtered pigs [29], suggesting a benefit for swine health.

The complete analysis of the OTUs revealed the ensemble of 18 phyla. The four prevalent phyla in all treatments were *Bacteroidetes*, *Firmicutes*, *Proteobacteria*, and *Spirochaetes*. The clustering using the Euclidean distance showed the clusterization between CuSO_4_ and TBCC and another cluster between Cu-MHAC and control samples (Fig. 1B, Table S2, and Fig. S2), as also found in a study that analyzed the cecal microbiota of piglets after antibiotic supplementation [41]. *Bacteroidetes* is a component of several microbiomes and the phylum is dominant in post-weaning piglet feces [15]. Their members are associated with the degradation of polysaccharides and proteins [42] contributing to gut homeostasis and health [43]. *Bacteroidetes* together with *Firmicutes* are related to obesity in humans [44, 45] and could be involved in the weight gain in swine.

Cu-MHAC showed (numerically) the lowest mean of *Proteobacteria* and *Spirochaetes* (approximately − 1.4 and − 2.1-fold compared to the other treatments), while TBCC showed the highest mean of both phyla (P > 0.05) (Table S2). A predominance of *Proteobacteria* has been reported in the wastewater of a farm environment, and its proportion was correlated to several disorders, including dysbiosis and inflammatory bowel disease (IBD), suggesting a close relationship between these bacteria and inflammation [46–50]. *Spirochaetes* includes a large group of motile bacteria with four clinically important genera: *Treponema*, *Borrelia*, *Leptospira*, and *Brachyspira.* They are disease agents for syphilis and Lyme disease. Bacteria included in this phylum are common in pigs and adult chickens with colitis/typhlitis, diarrhea, poor growth rates, and weight loss [51, 52].

*Euryarchaeota* abundance was high in the Control group than in copper-treated animals (P = 0.0442 by orthogonal contrast) and numerically lower in Cu-MHAC treated animals (P = 0.0812). This phylum is one of the most prevalent in the gut of swine and, together with *Proteobacteria* and *Fusobacteria*, are known as harmful to the intestine [53, 54]. The occurrence of members from *Synergistetes* was also decreased in the Cu-MHAC treatment. The abundance of this phylum was decreased with the elevation of Cu^2+^ and Zn^2+^ concentrations in swine wastewater and it was apparently involved in crude fiber (CF) digestibility [55, 56].

The cluster of the twenty most prevalent genera together with the “Unassigned” bacteria formed 2 distinct groups: a cluster of Cu-MHAC and Control, and another of CuSO_4_ and TBCC. (Fig. 1C). These most prevalent genera have been reported in several studies showing implications for modification of metabolic sources or biological activities [41, 57, 58] (Table S3). Among them, *Prevotella* prevailed numerically (mean = 32.8%; P > 0.10) (Table 2), especially in Cu-MHAC. This is in agreement with previous studies that showed a high abundance of the genus in the pig colon [59] and cecum [60]. *Prevotella* includes species that can be associated with (*i*) inflammatory features [61], (*ii*) high dietary consumption of carbohydrates [62] and fibers [63], (*iii*) increasing glycogen storage and protection against glucose intolerance [64], and (*iv*) more prevalent in healthy than diarrheic fecal samples of pigs [65, 66]. Therefore, the prevalence of this genus in the cecal microbiota can offer advantages to the health of piglets. In addition, in a recent study of our group, we showed that Cu-MHAC supplementation is accompanied by a significant increase of ghrelin mRNA in pigs [26]. The increase in ghrelin likely a stimulator of GH secretion thus acting as a positive factor in the performance of pigs and increasing weight gain [26]. Consistently, Queipo-Ortuño et al. found a relationship between gut microbiota and appetite-regulating hormones that associates ghrelin levels with an increase in the Prevotella genus in rats’ gut [67]. Considering that the piglets employed in this study had been also studied by Gonzalez-Esquerra [26], our results strongly supports the same relationship observed in rats. The increase of ghrelin and *Prevotella* phyla observed in the Cu-MHAC treatment was also important for a better feed conversion ratio (FCR) in our pigs. Additionally, the Cu-MHAC treatment also showed a numerical enrichment (Fig. 1C) in *Oscillibacter* (mean = 2.53%; P = 0.1450) which is a genus that has a recognized role in anti-inflammatory metabolites production [68].

Using 16 S rRNA sequencing, we were unable to differentiate between the Escherichia and Shigella genera since their V3-V4 sequences are identical. Although a previous study reported a decrease in Escherichia coli persistence after piglets received CuSO4 (175 mg kg-1) [69]. Our results showed a different pattern, since the category in which Escherichia and Shigella were grouped increased in all Cu treatments as shown by orthogonal contrast (P = 0.0245). Interestingly, the highest mean (mean = 3.8026) was recorded in the TBCC group (Table 2). Thus, because the 16 S analysis bias, this enrichment could be caused by an increase in Shigella or other Escherichia species that are commonly related to enteric diseases [70, 71]. Nonetheless, further studies are needed to clarify this point.

CuSO_4_ showed numerical enrichment in (*i*) *Helicobacter* (mean = 0.1985; P = 0.1110) (Fig. 1C) includes pathogenic members for animals and humans [72] causing colitis [47]; (*ii*) *Roseburia* (mean = 3.0873; P = 0.2500) that correlates with anti-inflammatory properties due to SCFAs production [73]; and (*iii*) *Succinivibrio* (mean = 5.9400; P = 0.2242), which produces succinate [74] and is a potential fiber-degrader [75]. Meanwhile, the Control had increased (Table 2) (*i*) *Megasphaera* (mean = 1.6950; P = 0.0686), which produces amino acids and vitamins [76], and (*ii*) *Paracteroides* (mean = 2.7010) and *Bacteroides* (mean = 1.5775; P = 0.2582), both bacteriocin producers that can protect the gut against exogenous microorganisms [77].

### The administration of different copper sources influenced the specific modulation of *Clostridium XIVa*, *Desulfovibrio*, and *Megasphera*

The 20 most prevalent genera were compared among Cu treated and control groups, taking into account the trial and treatment. The results showed that nine bacterial groups (*Prevotella, Treponema, Clostridium, Desulfovibrio, Megasphera, Streptococcus, Roseburia, Acidominococcus*, and *Escherichia/Shigella*) were differentially regulated (P < 0.05) between the two different trials. This indicates an impact of the environment on the microbiota since trials were done in different seasons. However, *Clostridium XIVa, Desulfovibrio*, and *Megasphera* had a regulation by treatment or source of Cu. *Desulfovibrio* increased when higher levels of Cu were used (P < 0.05 by contrast). In addition, CuSO_4_, Cu-MHAC, and TBCC increased *Desulfovibrio* prevalence compared to the Control (P < 0.001). An increment in the cluster *Clostridium XIVa* was observed only when the organic source or TBCC was used instead of CuSO_4_ (P < 0.05). For this cluster of bacteria, high levels of CuSO_4_ seem to have a role in the reduction of these microorganisms, since their abundance was lower and not different from the Control (Table 2). *Megasphera* (P = 0.0686) abundance was reduced by supra-nutritional levels of Cu (P < 0.05 by contrast).

**Table 2:** Comparison of the relative abundance means among the top 20 genera by treatment

Treatment	Genus						
	*Prevotella*	*Treponema*	*Bacteroides*	*Oscillibacter* ^*1*^	*Clostridium_XlVa*	*Desulfovibrio*	*Parabacteroides*
Control	29.8750	0.8400	1.5775	1.8825	1.1975	0.4638	2.7010
Cu-MHAC	32.7500	0.7343	0.4158	2.5275	1.4253	0.7847	0.5435
CuSO_4_	30.1500	1.4365	0.6033	2.4675	0.6033	0.5585	0.9645
TBCC	28.8250	1.4660	0.5840	1.2625	1.6445	0.6310	0.4895
CV_mean_ (%)	8.1	65.8	182.0	0.145	44.7	14.3	
***P*** **-value**							
Trial	< 0.0001**	0.0086**	0.1741	-	<0.0001**	<0.0001**	0.0982*
Treatment	0.2009	0.1291	0.9817	0.1450	0.0207**	0.0006*	0.2582
***P*** **-value for Orthogonal contrasts**							
Control x All	0.6330	0.3315	0.9809	-	0.2251	0.0013**	0.0589*
Cu-MHAC vs. (CuSO_4_ + TBCC)	0.6814	0.5422	0.9995	-	0.0044**	0.0033**	0.6705
CuSO_4_ vs. TBCC	0.0460**	0.0373**	0.6903	-	0.5215	0.0042**	0.9644
**Treatment**	**Genus**						
	*Clostridium_sensu_* *stricto*	*Succinivibrio*	*Campylobacter*	*Fusobacterium* ^*1*^	*Alloprevotella*	*Megasphaera*	*Streptococcus*
Control	1.2180	3.2515	1.6853	0.0755	5.0100	1.6950	0.5623
Cu-MHAC	1.1588	2.6115	1.6738	0.0260	3.2875	1.1105	0.7545
CuSO_4_	1.2220	5.9400	0.5828	0.7720	3.2275	1.0575	0.9475
TBCC	0.8010	4.0150	1.9038	0.4353	5.6900	0.9105	0.9150
CV_mean_ (%)	50.2	55.9	142.2	0.615	73.4	32.6	88.4
***P*** **-value**							
Trial	0.4086	0.1974	0.0091**	-	0.0016**	0.2694	0.0001**
Treatment	0.6702	0.2242	0.7304	0.6150	0.1038	0.0686*	0.6839
***P*** **-value for Orthogonal contrasts**							
Control x All	0.6315	0.4784	0.4870	-	0.8135	0.0125**	0.7089
Cu-MHAC vs. (CuSO_4_ + TBCC)	0.4892	0.0786*	0.9196	-	0.8283	0.8470	0.2664
CuSO_4_ vs. TBCC	0.3796	0.3888	0.3944	-	0.0183**	0.4819	0.9192
**Treatment**	**Genus**						
	*Roseburia*	*Actinobacillus*	*Helicobacter*	*Phascolarctobacterium*	*Acidaminococcus*	*Escherichia /Shigella*	
Control	1.9198	0.1473	0.0933	2.0000	1.0195	2.3318	
Cu-MHAC	1.4760	0.2163	0.0355	2.8350	0.8313	3.2350	
CuSO_4_	3.0873	0.5953	0.1985	2.5850	0.5483	3.6100	
TBCC	1.8483	0.2998	0.1353	2.8750	0.4458	3.8026	
CV_mean_ (%)	40.6	166.4	97.7	29.8	53.0	60.2	
***P*** **-value**							
Trial	<0.000**	0.0235**	0.3746	0.2148	0.0021**	0.0053**	
Treatment	0.2500	0.7069	0.1110	0.3879	0.3475	0.1279	
***P*** **-value for Orthogonal contrasts**							
Control x All	0.4989	0.3105	0.7716	0.1121	0.2960	0.0245**	
Cu-MHAC vs. (CuSO_4_ + TBCC)	0.0672*	0.7067	0.0405*	0.5771	0.8475	0.6287	
CuSO_4_ vs. TBCC	0.7312	0.7139	0.1733	0.9425	0.1476	0.6391	

Significance level was considered p-value < 0.05**, and p-value from 0.05 to 0.10 indicated a statistical trend*. Coefficient of variation of the mean (CV_mean_)

1 Freedman test

*Desulfovibrio* strains play an important role in the growth performance and health improvement of piglets during the early-weaned stage (Table S4). An increase in this group of bacteria was associated with a reduction in gut colonization of pathogens and an increase in energy conversion [79]. Additionally, *Desulfovibrio* was involved in the removal of the excess hydrogen generated by the microbiota during digestion, and this withdrawal has also been associated with the improvement of continuous SCFA production that was inhibited by an excess of hydrogen [80].

*Clostridium XIVa*, also known as *Clostridium coccoides* group, is a group of microorganisms that help the host to use nutrients that cannot be properly digested. These bacteria are also known as good SCFAs producers, playing an important role in intestinal homeostasis [81]. Shi et al. in observed an increase in these bacteria in the group of piglets supplemented with early food introduction (milk) that had an increase in SCFA production. SCFAs could contribute to the decrease of pro-inflammatory cytokines reducing the proliferation of pathogens [82] and contributing to the health of piglets (Table S4).

The homeostasis of the gut is a combination of several factors as the production of metabolites from both the host and its microbiota as well as the interaction of these metabolites between them. SCFAs have been reported to affect appetite regulation and energy homeostasis [83] and the gut microbiota-derived acetate was described as stimulating ghrelin secretion [84]. Gonzalez-Esquerra et al. [26] showed that the individuals used in this study and treated with Cu-MHAC had an increase in weight gain and the expression of mRNA for Ghrelin when compared to TBCC treatment. *Prevotella* is another genus involved in SCFA production, which was numerically increased in the group treated with Cu-MHAC. Finally, *Megasphaera* decreased after the Cu-MHAC treatment. These bacteria were usually found with an increment in the intestines of pigs in response to *Lactobacillus* and were correlated with intestinal disorders or immune responses in pigs. Additionally, they could be increased by the mycotoxins Deoxynivalenol (DON) and zearalenone (ZEN) which are frequently increased in the gut by ingestion of contaminated maize and grain cereals. Altogether, these results showed that different sources of copper in the pig diet promoted the proliferation of different genera of bacteria and that the interaction of the microbiota and the host could stimulate several genetic factors involved in gut health that could also modulate feed efficiency (FE), an important variable to swine production (Table S4).

## Conclusions

Different copper sources can modulate the cecal microbiota even of healthy piglets fed with antibiotics as growth promoters. Dominance and Simpson ecological indexes usually differed, showing a prevalence of specific genera according to treatment. Some groups of bacteria seem to be directly affected by the source of copper that was offered to the animals. Overall, Cu-MHAC can be a beneficial supplement even in low doses, since it seems to affect the diversity of bacteria, mainly *Desulfovibrio* and *Clostridium_XIVa* that are involved in the improvement of gut health and FE and SCFAs production (Table S4). The increase in SCFAs, especially acetate, could be involved in the modulation of ghrelin and growth hormone expression on top of improving performance as previously reported by our group [26]. A graphical abstract summarizing the methods and main results is shown in Fig S3.

## Electronic supplementary material

Below is the link to the electronic supplementary material.

Supplementary Material 2

Supplementary Material 3

## Data Availability

All data and material are available in the manuscript and as supplementary.

## List of abbreviations

Cu: copper
CuSO_4_: Copper (II) sulfate
TBCC: tri-basic copper chloride
Cu-MHAC: Cu methionine hydroxy analogue chelated
OTUs: Operational Taxonomic Units
SCFA: short chain fatty acids
FE: feed efficiency
GIT: gastrointestinal tract
Zn: zinc
ZnO: zinc oxide
DON: Deoxynivalenol
ZEN: zearalenone
FE: feed efficiency
ANOVA: Analysis of variance
CV_mean_: coefficient of variation of the mean
