# Supplementary material for: Assessment of cecal microbiota modulation from piglet dietary supplementation with copper
Source: BMC Microbiol. 2023 Mar 31;23:92. doi: 10.1186/s12866-023-02826-9 (PMC10064724; doi:10.1186/s12866-023-02826-9)
Supplement: Supplementary file 1 — Supplementary Material 2 [file 12866_2023_2826_MOESM2_ESM.docx]

**Assessment of cecal microbiota modulation from dietary supplementation with copper sources in piglets**


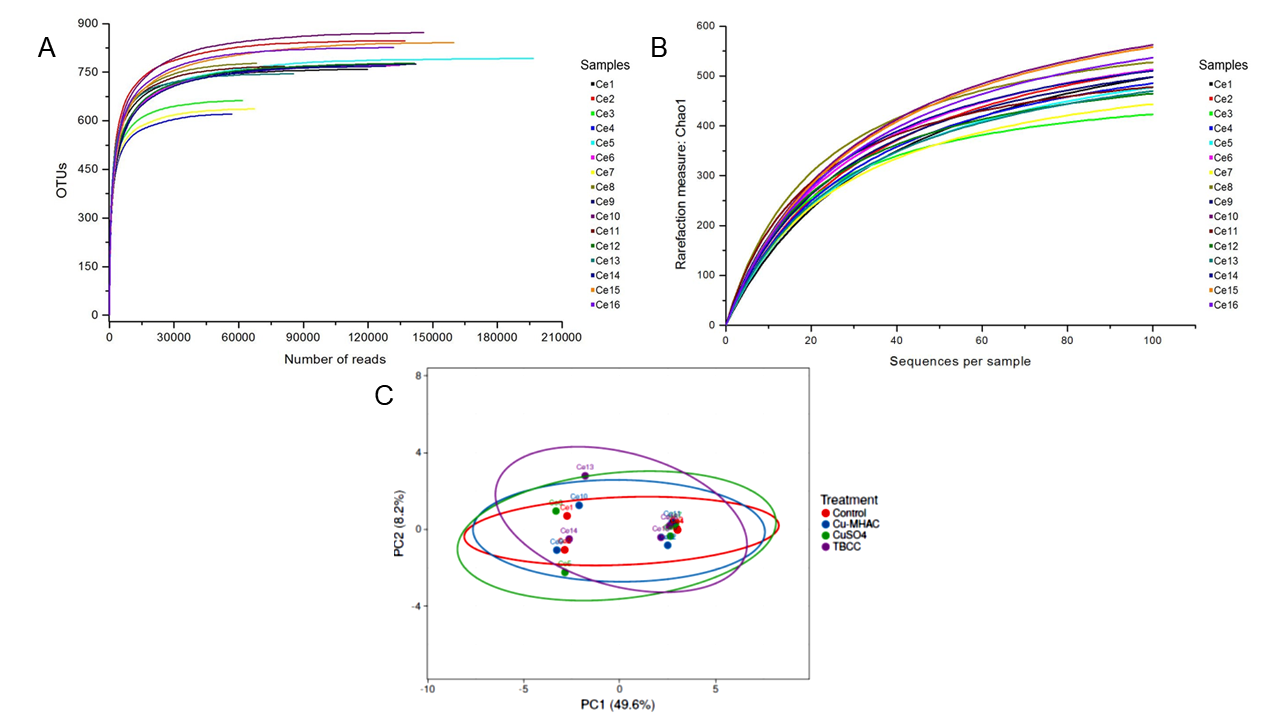


**FIG S1 Rarefaction and diversity analyzes.** (A) Rarefaction curve to the relation between the number of reads and OTUs by sample. (B) Rarefaction curve based on Chao1 metric estimated bacterial richness. (C) Principal component analysis (PCA) plot of beta diversity based on Bray-Curtis dissimilarity. Treatments with respective samples: Control (Ce1, Ce2, Ce3, Ce4), CuSO_4_ (Ce5, Ce6, Ce7, Ce8), Cu-MHAC (Ce9, Ce10, Ce11, Ce12), and TBCC (Ce13, Ce14, Ce15, Ce16).


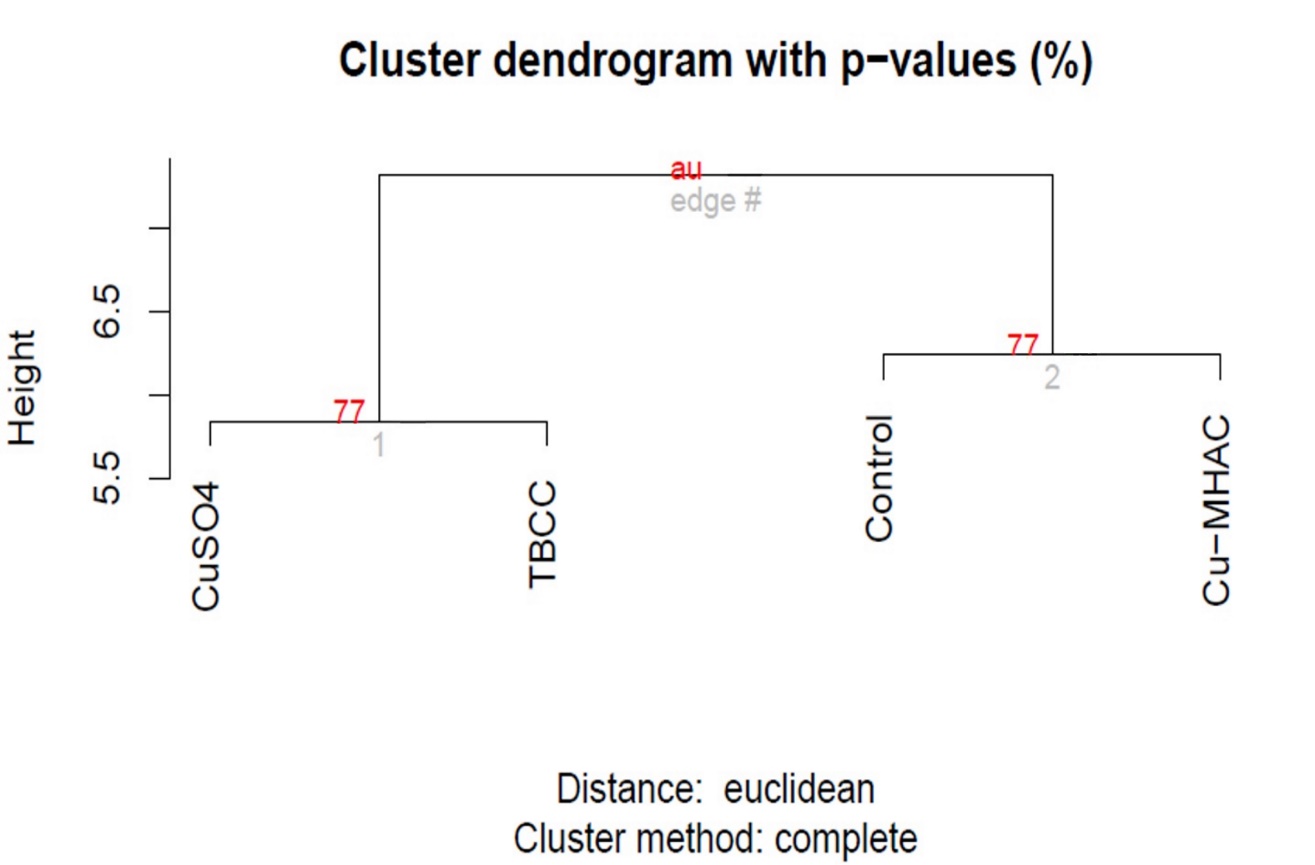


**FIG S2** **Hierarchical cluster analysis to the trials after 10000 interactions using Euclidian distance and complete method.**


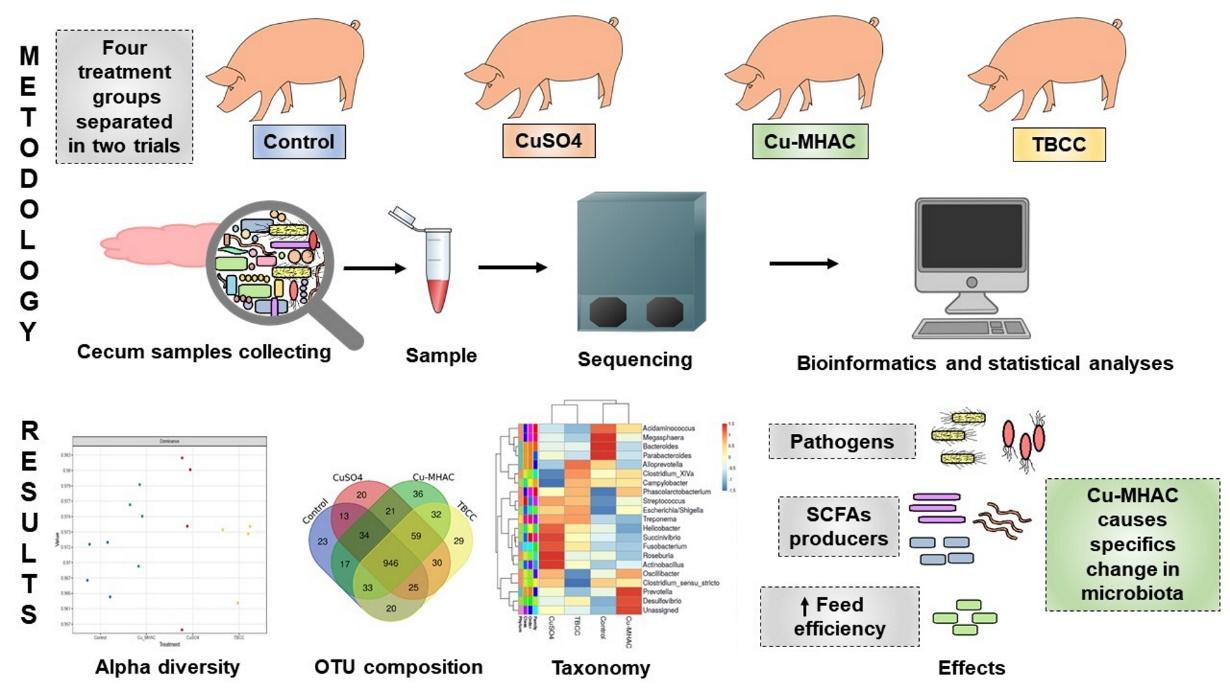


**FIG S3** The graphical abstract shows the methodology resume with the four treatments, the cecum contents collection, sequencing in Illumina MiSeq, and the analysis of sequencing. The main results were represented by graphics including the alpha diversity, OTU composition, and the most abundant genera. We represented some bacterias that are related to pathogenicity, SCFAs production, and FE increasing. Our conclusion about the Cu-MHAC was that it altered the cecum microbiota and could be used as an alternative supplement for swine, once the results were satisfactory.

**Table S1: Total number of OTUs and reads by sample.**

|  | **Treatment** | | | | | | | | | | | | | | | |
| --- | --- | --- | --- | --- | --- | --- | --- | --- | --- | --- | --- | --- | --- | --- | --- | --- |
|  | **Control** | | | | **CuSO_4_** | | | | **Cu-MHAC** | | | | **TBCC** | | | |
| **Sample** | **Ce1** | **Ce2** | **Ce3** | **Ce4** | **Ce5** | **Ce6** | **Ce7** | **Ce8** | **Ce9** | **Ce10** | **Ce11** | **Ce12** | **Ce13** | **Ce14** | **Ce15** | **Ce16** |
| **Reads** | 119699 | 137219 | 61773 | 56819 | 196645 | 134606 | 67520 | 68241 | 142206 | 145924 | 81161 | 141579 | 85401 | 128192 | 159922 | 131815 |
| **OTUs** | 759 | 847 | 662 | 620 | 792 | 771 | 637 | 777 | 775 | 872 | 768 | 777 | 745 | 768 | 841 | 826 |

**Table S2 Phyla relative abundance means by treatment and orthogonal contrast tests.**

| **Treatment** | **Phylum** | | | | | | | |  |
| --- | --- | --- | --- | --- | --- | --- | --- | --- | --- |
|  | **Unknown#** | **Synergistetes#** | **Euryarchaeota#** | **Firmicutes** | **Bacteroide-**  **tes** | **Proteobac-**  **teria** | **Spirochaetes#** | **Actinobac-**  **teria#** | **Tenericutes#** |
| Control | 5.6450 | 0.0285 | 0.4773 | 28.4750 | 49.7750 | 13.6000 | 1.3700 | 0.0150 | 0.1683 |
| Cu-MHAC | 5.1050 | 0.0248 | 0.1636 | 31.0000 | 50.9500 | 11.3950 | 0.9325 | 0.0329 | 0.1296 |
| CuSO4 | 4.0050 | 0.0463 | 0.3235 | 31.1000 | 47.9750 | 13.6175 | 1.8150 | 0.0205 | 0.1384 |
| TBCC | 3.2750 | 0.0184 | 0.2636 | 29.3750 | 48.8750 | 15.3500 | 2.0775 | 0.0248 | 0.2036 |
| CVmean (%) | 0.6001 | 0.0083 | 0.1011 | 1.1702 | 1.3211 | 1.2255 | 0.4528 | 0.0056 | 0.0690 |
| P-value for ANOVA |  |  |  |  |  |  |  |  |  |
| Trial | 0.0524* | 0.0016** | <0.0001** | 0.0581* | 0.1260 | 0.0004** | 0.1402 | 0.0019** | 0.0226** |
| Treatment | 0.0558* | 0.1230 | 0.0812* | 0.3554 | 0.4603 | 0.2150 | 0.3423 | 0.2040 | 0.8696 |
| P-value for Orthogonal contrasts |  |  |  |  |  |  |  |  |  |
| Control vs Others | 0.0745* | 0.9065 | 0.0442** | 0.1637 | 0.7452 | 0.9198 | 0.8802 | 0.2066 | 0.7256 |
| Cu-MHAC vs  (CuSO4 +TBCC) | 0.8876 | 0.0330** | 0.1969 | 0.5374 | 0.2563 | 0.8733 | 0.7011 | 0.0631* | 0.8266 |
| CuSO4 vs TBCC | 0.2787 | 0.1497 | 0.5910 | 0.3196 | 0.6394 | 0.3390 | 0.3502 | 0.1764 | 0.9047 |

Significance level was considered p-value < 0.05**, and p-value from 0.05 to 0.10 indicated a statistical trend*. Coefficient of variation of the mean (CVmean).

# Data transformed to log

**Table S3 Genus relative abundances means by treatment and the taxonomic levels correspondents.**

| **Taxonomy** | | | | | **Treatment** | | | |
| --- | --- | --- | --- | --- | --- | --- | --- | --- |
| **Phylum** | **Class** | **Order** | **Family** | **Genus** | **Control** | **Cu-MHAC** | **CuSO_4_** | **TBCC** |
| Bacteroidetes | Bacteroidia | Bacteroidales | Prevotellaceae | Prevotella | 29.8750 | 32.7500 | 30.1500 | 28.8250 |
| Spirochaetes | Spirochaetia | Spirochaetales | Spirochaetaceae | Treponema | 0.8400 | 0.7343 | 1.4365 | 1.4660 |
| Bacteroidetes | Bacteroidia | Bacteroidales | Bacteroidaceae | Bacteroides | 1.5775 | 0.4158 | 0.6033 | 0.5840 |
| Firmicutes | Clostridia | Clostridiales | Ruminococcaceae | Oscillibacter | 1.8825 | 2.5275 | 2.4675 | 1.2625 |
| Firmicutes | Clostridia | Clostridiales | Lachnospiraceae | Clostridium_XlVa | 1.1975 | 1.4253 | 0.6033 | 1.6445 |
| Proteobacteria | Deltaproteobacteria | Desulfovibrionales | Desulfovibrionaceae | Desulfovibrio | 0.4638 | 0.7847 | 0.5585 | 0.6310 |
| Bacteroidetes | Bacteroidia | Bacteroidales | Porphyromonadaceae | Parabacteroides | 2.7000 | 0.5400 | 0.9600 | 0.4900 |
| Firmicutes | Clostridia | Clostridiales | Clostridiaceae | Clostridium_sensu_stricto | 1.2180 | 1.1588 | 1.2220 | 0.8010 |
| Proteobacteria | Gammaproteobacteria | Aeromonadales | Succinivibrionaceae | Succinivibrio | 3.2515 | 2.6115 | 5.9400 | 4.0150 |
| Proteobacteria | Epsilonproteobacteria | Campylobacterales | Campylobacteraceae | Campylobacter | 1.6853 | 1.6738 | 0.5828 | 1.9038 |
| Fusobacteria | Fusobacteriia | Fusobacteriales | Fusobacteriaceae | Fusobacterium | 0.0755 | 0.0260 | 0.7720 | 0.4353 |
| Bacteroidetes | Bacteroidia | Bacteroidales | Prevotellaceae | Alloprevotella | 5.0100 | 3.2875 | 3.2275 | 5.6900 |
| Firmicutes | Negativicutes | Selenomonadales | Veillonellaceae | Megasphaera | 1.6950 | 1.1105 | 1.0575 | 0.9105 |
| Firmicutes | Bacilli | Lactobacillales | Streptococcaceae | Streptococcus | 0.5623 | 0.7545 | 0.9475 | 0.9150 |
| Firmicutes | Clostridia | Clostridiales | Lachnospiraceae | Roseburia | 1.9198 | 1.4760 | 3.0873 | 1.8483 |
| Proteobacteria | Gammaproteobacteria | Pasteurellales | Pasteurellaceae | Actinobacillus | 0.1473 | 0.2163 | 0.5953 | 0.2998 |
| Proteobacteria | Epsilonproteobacteria | Campylobacterales | Helicobacteraceae | Helicobacter | 0.0933 | 0.0355 | 0.1985 | 0.1353 |
| Firmicutes | Negativicutes | Selenomonadales | Acidaminococcaceae | Phascolarctobacterium | 2.0000 | 2.8350 | 2.5850 | 2.8750 |
| Firmicutes | Negativicutes | Selenomonadales | Acidaminococcaceae | Acidaminococcus | 1.0195 | 0.8313 | 0.5483 | 0.4458 |
| Proteobacteria | Gammaproteobacteria | Enterobacteriales | Enterobacteriaceae | Escherichia/Shigella | 2.3318 | 3.2350 | 3.6100 | 3.8026 |
| Others | Others | Others | Others | Unassigned | 33.4500 | 37.5750 | 35.2000 | 34.4000 |

**TABLE S4** **Genus enriched by treatment and functional correlations.**

| **Treatment** | **Genus** | **Metabolic sources** | **Biological activities** |
| --- | --- | --- | --- |
| CuSO_4_ | *Streptococcus* | Amino acids metabolism (1) and acetate producer (2) | Diseases potential members (3), as *S. suis* is a common pathogen in pigs (4) |
|  | *Helicobacter* |  | Modify the epithelial proliferation and E-cadherin expression in pig gut mucosa (5) and related to gastric disorders (6) |
|  | *Fusobacterium* | Butyrate producer (7) | Some species implicated in pathologies (8) |
|  | *Roseburia* | Acetate. propionate and butyrate producer (9, 10) | Support immune defense (10) |
|  | *Faecalibacterium* | Butyrate and acetate producer (9, 11) | Decreasing in gut disorders (12) |
|  | *Actinobacillus* | Succinic acid producer (13) | Pathogenic members (14) |
| TBCC | *Alloprevotella* | Acetic and succinic acid producer (15) |  |
|  | *Clostridium_XIVa* | Butyrate producer (16) |  |
|  | *Campylobacter* |  | Pathogenic species (17) |
|  | *Escherichia/Shigella* | *E.coli* is associated with phenol production (18) | Pathogenic members (19, 20) |
|  | *Treponema* |  |  |
| Control | *Acidaminococcus* | Acetic and butyric acids and CO_2_ producer (21) |  |
|  | *Megasphaera* | Acetic, propionic, butyric, and valeric acid (22), amino acids, vitamins, acetate and butyrate producer (23) |  |
|  | *Bacteroides* | Breakdown polysaccharides (24), propionate and acetate producer (2) | Bacteriocin producer (25) |
|  | *Parabacteroides* | *P. distasonis* generates succinate and secondary bile acids in the gut (26) | Bacteriocins producer (25) |
|  |  |  |  |
| Cu-MHAC | *Oscillibacter* |  | Anti-inflammatory metabolites producer (27) |
|  | *Prevotella* | Acetate (2) and propionate (27) producer |  |
|  | *Desulfovibrio* | Sulphate-reducing (28) | Related to inflammatory bowel disease (28) |

**REFERENCES TABLE S4**

1. Dai ZL. Wu G. Zhu WY. 2011. Amino acid metabolism in intestinal bacteria: Links between gut ecology and host health. Front Biosci 16:1768–1786.

2. Feng W. Ao H. Peng C. 2018. Gut microbiota. short-chain fatty acids. and herbal medicines. Front Pharmacol 9:1354.

3. Krzyaściak W. Pluskwa KK. Jurczak A. Koaścielniak D. 2013. The pathogenicity of the Streptococcus genus. Eur J Clin Microbiol Infect Dis 32:1361–1376.

4. Murase K. Watanabe T. Arai S. Kim H. Tohya M. Ishida-Kuroki K. Võ TH. Nguy TPB. Nakagawa I. Osawa R. Nguyn NH. Sekizaki T. 2019. Characterization of pig saliva as the major natural habitat of Streptococcus suis by analyzing oral. fecal. vaginal. and environmental microbiota. PLoS One 14:e0215983.

5. Bracarense APFRL. Yamasaki L. Silva EO. Oliveira RL. Alfieri AA. 2013. Helicobacter spp. infection induces changes in epithelial proliferation and E-cadherin expression in the gastric mucosa of pigs. J Comp Pathol 149:402–409.

6. Mladenova-Hristova I. Grekova O. Patel A. 2017. Zoonotic potential of Helicobacter spp. J Microbiol Immunol Infect 50:265–269.

7. Anand S. Kaur H. Mande SS. 2016. Comparative In silico Analysis of Butyrate Production Pathways in Gut Commensals and Pathogens. Front Microbiol 7:1945.

8. McGuire AM. Cochrane K. Griggs AD. Haas BJ. Abeel T. Zeng Q. Nice JB. Macdonald H. Birren BW. Berger BW. Allen-Vercoe E. Earl AM. 2014. Evolution of invasion in a diverse set of Fusobacterium species. MBio 5:e01864-14.

9. Duncan SH. Barcenilla A. Stewart CS. Pryde SE. Flint HJ. 2002. Acetate utilization and butyryl coenzyme A (CoA): Acetate-CoA transferase in butyrate-producing bacteria from the human large intestine. Appl Environ Microbiol 68:5186–5190.

10. Tamanai-Shacoori Z. Smida I. Bousarghin L. Loreal O. Meuric V. Fong SB. Bonnaure-Mallet M. Jolivet-Gougeon A. 2017. Roseburia spp.: A marker of health? Future Microbiol 12:157–170.

11. Tremaroli V. Bäckhed F. 2012. Functional interactions between the gut microbiota and host metabolism. Nature 489:242–249.

12. Lopez-Siles M. Duncan SH. Garcia-Gil LJ. Martinez-Medina M. 2017. Faecalibacterium prausnitzii: From microbiology to diagnostics and prognostics. ISME J 11:841–852.

13. Bradfield MFA. Mohagheghi A. Salvachúa D. Smith H. Black BA. Dowe N. Beckham GT. Nicol W. 2015. Continuous succinic acid production by Actinobacillus succinogenes on xylose-enriched hydrolysate. Biotechnol Biofuels 8:181.

14. Ke S. Fang S. He M. Huang X. Yang H. Yang B. Chen C. Huang L. 2019. Age-based dynamic changes of phylogenetic composition and interaction networks of health pig gut microbiome feeding in a uniformed condition. BMC Vet Res 15:172.

15. Downes J. Dewhirst FE. Tanner ACR. Wade WG. 2013. Description of Alloprevotella rava gen. nov.. sp. nov.. isolated from the human oral cavity. and reclassification of Prevotella tannerae Moore et al. 1994 as Alloprevotella tannerae gen. nov.. comb. nov. Int J Syst Evol Microbiol 63:1214–1218.

16. Ríos-Covián D. Ruas-Madiedo P. Margolles A. Gueimonde M. De los Reyes-Gavilán CG. Salazar N. 2016. Intestinal short chain fatty acids and their link with diet and human health. Front Microbiol 7:185.

17. Epps S. Harvey R. Hume M. Phillips T. Anderson R. Nisbet D. 2013. Foodborne Campylobacter: Infections. Metabolism. Pathogenesis and Reservoirs. Int J Environ Res Public Health 10:6292–6304.

18. Kim B. Park H. Na D. Lee SY. 2014. Metabolic engineering of Escherichia coli for the production of phenol from glucose. Biotechnol J 9:621–629.

19. Khalil IA. Troeger C. Blacker BF. Rao PC. Brown A. Atherly DE. Brewer TG. Engmann CM. Houpt ER. Kang G. Kotloff KL. Levine MM. Luby SP. MacLennan CA. Pan WK. Pavlinac PB. Platts-Mills JA. Qadri F. Riddle MS. Ryan ET. Shoultz DA. Steele AD. Walson JL. Sanders JW. Mokdad AH. Murray CJL. Hay SI. Reiner RC. 2018. Morbidity and mortality due to shigella and enterotoxigenic Escherichia coli diarrhoea: the Global Burden of Disease Study 1990–2016. Lancet Infect Dis 18:1229–1240.

20. Dos Reis RS. Horn F. 2014. Enteropathogenic Escherichia coli. Samonella. Shigella and Yersinia: Cellular aspects of host-bacteria interactions in enteric diseases. Gut Pathog 2:8.

21. Rogosa M. 1969. Acidaminococcus gen. n.. Acidaminococcus fermentans sp. n.. anaerobic gram-negative diplococci using amino acids as the sole energy source for growth. J Bacteriol 98:756–766.

22. Marchandin H. Juvonen R. Haikara A. 2015. *Megasphaera*. p. 1–16. *In* Bergey’s Manual of Systematics of Archaea and Bacteria. John Wiley & Sons. Ltd. Chichester. UK.

23. Shetty SA. Marathe NP. Lanjekar V. Ranade D. Shouche YS. 2013. Comparative Genome Analysis of Megasphaera sp. Reveals Niche Specialization and Its Potential Role in the Human Gut. PLoS One 8:e79353.

24. Schwalm ND. Groisman EA. 2017. Navigating the Gut Buffet: Control of Polysaccharide Utilization in Bacteroides spp. Trends Microbiol 25:1005–1015.

25. Nakano V. Ignacio A. Fernandes MR. Fukugaiti MH. Avila-campos MJ. 2013. Intestinal Bacteroides and Parabacteroides species producing antagonistic substances. Curr Trends Microbiol 1:1–4.

26. Wang K. Liao M. Zhou N. Bao L. Ma K. Zheng Z. Wang Y. Liu C. Wang W. Wang J. Liu SJ. Liu H. 2019. Parabacteroides distasonis Alleviates Obesity and Metabolic Dysfunctions via Production of Succinate and Secondary Bile Acids. Cell Rep 26:222-235.e5.

27. Li J. Sung CYJ. Lee N. Ni Y. Pihlajamäki J. Panagiotou G. El-Nezami H. 2016. Probiotics modulated gut microbiota suppresses hepatocellular carcinoma growth in mice. Proc Natl Acad Sci U S A 113:E1306–E1315.

28. Chen Y ‐R.. Zhou L ‐Z.. Fang S ‐T.. Long H ‐Y.. Chen J ‐Y.. Zhang G ‐X. 2019. Isolation of *Desulfovibrio* spp. from human gut microbiota using a next‐generation sequencing directed culture method. Lett Appl Microbiol 68:553–561.
