# Supplementary material for: Assessment of cecal microbiota modulation from piglet dietary supplementation with copper
Source: BMC Microbiol. 2023 Mar 31;23:92. doi: 10.1186/s12866-023-02826-9 (PMC10064724; doi:10.1186/s12866-023-02826-9)
Supplement: Supplementary file 2 — Supplementary Material 3 [file 12866_2023_2826_MOESM3_ESM.docx]

**Table 1 Alpha diversity metrics means by treatment, ANOVA, and orthogonal contrasts analysis.**

| **Treatment** | **Alpha diversity metrics** | | | | | | | | | | |
| --- | --- | --- | --- | --- | --- | --- | --- | --- | --- | --- | --- |
|  | Berger Parker | Buzas Gibson | Chao 1 | Dominance | Equitability | Jost | Jost 1 | Richness | Robbins | Shannon | Simpson |
| Control | 0.1190 | 0.0011 | 723.2250 | 0.9692 | 0.6827 | 49.1000 | 88.9250 | 722.0000 | 0.1607 | 1.9475 | 0.0306 |
| Cu-MHAC | 0.0998 | 0.0008 | 199.1000 | 0.9750 | 0.6957 | 59.6000 | 104.7500 | 798.0000 | 0.1400 | 2.0150 | 0.0250 |
| CuSO4 | 0.0968 | 0.0011 | 745.4000 | 0.9787 | 0.6940 | 59.4500 | 101.0750 | 744.2500 | 0.1477 | 1.9900 | 0.0212 |
| TBCC | 0.1212 | 0.0008 | 796.0500 | 0.9700 | 0.6832 | 51.8500 | 96.7000 | 795.0000 | 0.1367 | 1.9825 | 0.0301 |
| CVmean (%) | 30.7300 | 38.1200 | 8.7000 | 0.5000 | 3.2600 | 21.6200 | 17.0200 | 8.7400 | 17.6000 | 3.6300 | 17.9800 |
| P-value for ANOVA |  |  |  |  |  |  |  |  |  |  |  |
| Trial | 0.5294 | 0.0167** | 0.1433 | 0.9306 | 0.0689* | 0.4072 | 0.2818 | 0.1436 | 0.0434** | 0.2635 | 0.8741 |
| Treatment | 0.6451 | 0.4934 | 0.3300 | 0.0908* | 0.7711 | 0.5155 | 0.5890 | 0.3313 | 0.5784 | 0.6298 | 0.0892* |
| P-value for Orthogonal contrasts |  |  |  |  |  |  |  |  |  |  |  |
| Control vs Others | 0.5141 | 0.3868 | 0.1666 | 0.0920* | 0.5374 | 0.2762 | 0.2410 | 0.1671 | 0.2219 | 0.2703 | 0.0989* |
| Cu-MHAC vs (CuSO4 +TBCC) | 0.5177 | 0.2288 | 0.2272 | 0.0916* | 0.7495 | 0.6191 | 0.9732 | 0.2281 | 0.5642 | 0.8466 | 0.0867* |
| CuSO4 vs TBCC | 0.3860 | 0.7304 | 0.9495 | 0.1772 | 0.4476 | 0.3765 | 0.5084 | 0.9505 | 0.8616 | 0.5370 | 0.1676 |

Significance level was considered p-value < 0.05**, and p-value from 0.05 to 0.10 indicated a statistical trend*. Coefficient of variation of the mean (CVmean).

**Table 2. Comparison of the relative abundance means among the top 20 genera by treatment**

| **Treatment** | **Genus** | | | | | | |
| --- | --- | --- | --- | --- | --- | --- | --- |
|  | **Prevotella** | **Treponema** | **Bacteroides** | **Oscillibacter1** | **Clostridium_XlVa** | **Desulfovibrio** | **Parabacteroides** |
| Control | 29.8750 | 0.8400 | 1.5775 | 1.8825 | 1.1975 | 0.4638 | 2.7010 |
| Cu-MHAC | 32.7500 | 0.7343 | 0.4158 | 2.5275 | 1.4253 | 0.7847 | 0.5435 |
| CuSO4 | 30.1500 | 1.4365 | 0.6033 | 2.4675 | 0.6033 | 0.5585 | 0.9645 |
| TBCC | 28.8250 | 1.4660 | 0.5840 | 1.2625 | 1.6445 | 0.6310 | 0.4895 |
| CVmean (%) | 8.1 | 65.8 | 182.0 | 0.145 | 44.7 | 14.3 |  |
| P-value |  |  |  |  |  |  |  |
| Trial | <0.0001** | 0.0086** | 0.1741 | - | <0.0001** | <0.0001** | 0.0982* |
| Treatment | 0.2009 | 0.1291 | 0.9817 | 0.1450 | 0.0207** | 0.0006** | 0.2582 |
| P-value for Orthogonal contrasts |  |  |  |  |  |  |  |
| Control x All | 0.6330 | 0.3315 | 0.9809 | - | 0.2251 | 0.0013** | 0.0589* |
| Cu-MHAC vs (CuSO4 +TBCC) | 0.6814 | 0.5422 | 0.9995 | - | 0.0044** | 0.0033** | 0.6705 |
| CuSO4 vs TBCC | 0.0460** | 0.0373** | 0.6903 | - | 0.5215 | 0.0042** | 0.9644 |

Significance level was considered p-value < 0.05**, and p-value from 0.05 to 0.10 indicated a statistical trend*. Coefficient of variation of the mean (CVmean).

1 Freedman test

| **Treatment** | **Genus** | | | | | | |
| --- | --- | --- | --- | --- | --- | --- | --- |
|  | **Clostridium_sensu_**  **stricto** | **Succinivibrio** | **Campylobacter** | **Fusobacterium1** | **Alloprevotella** | **Megasphaera** | **Streptococcus** |
| Control | 1.2180 | 3.2515 | 1.6853 | 0.0755 | 5.0100 | 1.6950 | 0.5623 |
| Cu-MHAC | 1.1588 | 2.6115 | 1.6738 | 0.0260 | 3.2875 | 1.1105 | 0.7545 |
| CuSO4 | 1.2220 | 5.9400 | 0.5828 | 0.7720 | 3.2275 | 1.0575 | 0.9475 |
| TBCC | 0.8010 | 4.0150 | 1.9038 | 0.4353 | 5.6900 | 0.9105 | 0.9150 |
| CVmean (%) | 50.2 | 55.9 | 142.2 | 0.615 | 73.4 | 32.6 | 88.4 |
| P-value |  |  |  |  |  |  |  |
| Trial | 0.4086 | 0.1974 | 0.0091** | - | 0.0016** | 0.2694 | 0.0001** |
| Treatment | 0.6702 | 0.2242 | 0.7304 | 0.6150 | 0.1038 | 0.0686* | 0.6839 |
| P-value for Orthogonal contrasts |  |  |  |  |  |  |  |
| Control x All | 0.6315 | 0.4784 | 0.4870 | - | 0.8135 | 0.0125** | 0.7089 |
| Cu-MHAC vs (CuSO4 +TBCC) | 0.4892 | 0.0786* | 0.9196 | - | 0.8283 | 0.8470 | 0.2664 |
| CuSO4 vs TBCC | 0.3796 | 0.3888 | 0.3944 | - | 0.0183** | 0.4819 | 0.9192 |

Significance level was considered p-value < 0.05**, and p-value from 0.05 to 0.10 indicated a statistical trend*. Coefficient of variation of the mean (CVmean).

1 Freedman test

| **Treatment** | **Genus** | | | | | |
| --- | --- | --- | --- | --- | --- | --- |
|  | **Roseburia** | **Actinobacillus** | **Helicobacter** | **Phascolarctobacterium** | **Acidaminococcus** | **Escherichia /Shigella** |
| Control | 1.9198 | 0.1473 | 0.0933 | 2.0000 | 1.0195 | 2.3318 |
| Cu-MHAC | 1.4760 | 0.2163 | 0.0355 | 2.8350 | 0.8313 | 3.2350 |
| CuSO4 | 3.0873 | 0.5953 | 0.1985 | 2.5850 | 0.5483 | 3.6100 |
| TBCC | 1.8483 | 0.2998 | 0.1353 | 2.8750 | 0.4458 | 3.8026 |
| CVmean (%) | 40.6 | 166.4 | 97.7 | 29.8 | 53.0 | 60.2 |
| P-value |  |  |  |  |  |  |
| Trial | <0.000** | 0.0235** | 0.3746 | 0.2148 | 0.0021** | 0.0053** |
| Treatment | 0.2500 | 0.7069 | 0.1110 | 0.3879 | 0.3475 | 0.1279 |
| P-value for Orthogonal contrasts |  |  |  |  |  |  |
| Control x All | 0.4989 | 0.3105 | 0.7716 | 0.1121 | 0.2960 | 0.0245** |
| Cu-MHAC vs (CuSO4 +TBCC) | 0.0672* | 0.7067 | 0.0405* | 0.5771 | 0.8475 | 0.6287 |
| CuSO4 vs TBCC | 0.7312 | 0.7139 | 0.1733 | 0.9425 | 0.1476 | 0.6391 |

Significance level was considered p-value < 0.05**, and p-value from 0.05 to 0.10 indicated a statistical trend*. Coefficient of variation of the mean (CVmean).

1 Freedman test

**Table S2 Phyla relative abundance means by treatment and orthogonal contrast tests.**

| **Treatment** | **Phylum** | | | | | | | |  |
| --- | --- | --- | --- | --- | --- | --- | --- | --- | --- |
|  | **Unknown#** | **Synergistetes#** | **Euryarchaeota#** | **Firmicutes** | **Bacteroide-**  **tes** | **Proteobac-**  **teria** | **Spirochaetes#** | **Actinobac-**  **teria#** | **Tenericutes#** |
| Control | 5.6450 | 0.0285 | 0.4773 | 28.4750 | 49.7750 | 13.6000 | 1.3700 | 0.0150 | 0.1683 |
| Cu-MHAC | 5.1050 | 0.0248 | 0.1636 | 31.0000 | 50.9500 | 11.3950 | 0.9325 | 0.0329 | 0.1296 |
| CuSO4 | 4.0050 | 0.0463 | 0.3235 | 31.1000 | 47.9750 | 13.6175 | 1.8150 | 0.0205 | 0.1384 |
| TBCC | 3.2750 | 0.0184 | 0.2636 | 29.3750 | 48.8750 | 15.3500 | 2.0775 | 0.0248 | 0.2036 |
| CVmean (%) | 0.6001 | 0.0083 | 0.1011 | 1.1702 | 1.3211 | 1.2255 | 0.4528 | 0.0056 | 0.0690 |
| P-value for ANOVA |  |  |  |  |  |  |  |  |  |
| Trial | 0.0524* | 0.0016** | <0.0001** | 0.0581* | 0.1260 | 0.0004** | 0.1402 | 0.0019** | 0.0226** |
| Treatment | 0.0558* | 0.1230 | 0.0812* | 0.3554 | 0.4603 | 0.2150 | 0.3423 | 0.2040 | 0.8696 |
| P-value for Orthogonal contrasts |  |  |  |  |  |  |  |  |  |
| Control vs Others | 0.0745* | 0.9065 | 0.0442** | 0.1637 | 0.7452 | 0.9198 | 0.8802 | 0.2066 | 0.7256 |
| Cu-MHAC vs  (CuSO4 +TBCC) | 0.8876 | 0.0330** | 0.1969 | 0.5374 | 0.2563 | 0.8733 | 0.7011 | 0.0631* | 0.8266 |
| CuSO4 vs TBCC | 0.2787 | 0.1497 | 0.5910 | 0.3196 | 0.6394 | 0.3390 | 0.3502 | 0.1764 | 0.9047 |

Significance level was considered p-value < 0.05**, and p-value from 0.05 to 0.10 indicated a statistical trend*. Coefficient of variation of the mean (CVmean).

# Data transformed to log

**­Continuation**

**Table S2 Phyla relative abundance means by treatment and orthogonal contrast tests.**

| **Treatment** | **Phylum** | | | | | | | | | |
| --- | --- | --- | --- | --- | --- | --- | --- | --- | --- | --- |
|  | **Fusobac-teria** | **Lentis-phaerae** | **Verrucomi-crobia** | **Fibrobac-teres** | **Cyanobacteria_**  **Chloroplast** | **Candidatus_**  **Saccharibac-teria** | **Deferribac-teres** | **Chlamy-diae** | **Planctomy-cetes** | **Elusimi-crobia** |
| Control | 0.0760 | 0.0970 | 0.0240 | 0.0060 | 0.0040 | 0.0440 | 0.0320 | 0.0550 | 0.0030 | 0.0910 |
| Cu-MHAC | 0.0270 | 0.0270 | 0.0210 | 0.0070 | 0.0040 | 0.0230 | 0.0300 | 0.0880 | 0.0330 | 0.0350 |
| CuSO4 | 0.7810 | 0.0270 | 0.0100 | 0.0160 | 0.0020 | 0.0140 | 0.0080 | 0.0030 | 0.0040 | 0.0800 |
| TBCC | 0.4380 | 0.0260 | 0.0250 | 0.0170 | 0.0060 | 0.0260 | 0.0040 | 0.0400 | 0.0110 | 0.0280 |
| CVmean (%) | 0.3600 | 0.0229 | 0.1463 | 0.0046 | 0.0015 | 0.0155 | 0.0169 | 0.0430 | 0.0143 | 0.0472 |
| P-value for Freedman test |  |  |  |  |  |  |  |  |  |  |
| Trial |  |  |  |  |  |  |  |  |  |  |
| Treatment | 0.6150 | 0.7530 | 0.2410 | 0.3920 | 0.2410 | 0.6150 | 0.4940 | 0.2410 | 0.1750 | 0.4940 |

Significance level was considered p-value < 0.05**, and p-value from 0.05 to 0.10 indicated a statistical trend*. Coefficient of variation of the mean (CVmean).

# Data transformed to log
